# Tribological properties of high-speed steel surface with texture and vertical fibers

**DOI:** 10.1038/s41598-023-39721-2

**Published:** 2023-08-14

**Authors:** Kai Feng, Jing Ni, Zixuan Wang, Zhen Meng

**Affiliations:** School of Mechanical Engineering, Hangzhou Dianzi University, Hangzhou, 310018 China

**Keywords:** Mechanical engineering, Soft materials

## Abstract

Inadequate lubrication of the two touching surfaces during friction can lead to severe wear, especially in metal cutting. Therefore, a surface with synergistic anti-friction effect of texture and solid lubricant was proposed to improve lubrication. A mesh texture with excellent wettability was prepared on the high-speed steel (HSS) surface by laser, and then nylon fibers were vertically implanted into the grooves of the texture using the electrostatic flocking technology. The friction and wear state of different surfaces (smooth, textured, flocking) under dry/oil-lubricated were studied by a linear reciprocating wear tester. The coefficient of friction (COF) under different working conditions was used to analyze the anti-friction properties, and the wear rate was used to evaluate the wear resistance of the surface. The results showed that the tribological properties of flocking surfaces were better than those of the other two surfaces. This is because the addition of nylon fibers eases shear at the edges of the texture. The broken fibers form a solid lubricating film on the specimen surface, which prevents the surface from being scratched by debris. In addition, it is found that COF decreases with increasing load. Finally, the rapid wettability of the oil droplets on the flocking surface shows the great potential of the surface for lubrication and anti-friction.

## Introduction

Wear caused by friction is one of the main reasons for the failure of equipment and parts^1^. Therefore, the research on friction reduction has attracted the attention of many scholars, mainly focusing on new materials, surface texture, coatings, lubricants and other aspects. In the field of metal cutting processing, the material is violently rubbed with the tool surface, thus creating a high-temperature and high-pressure area at the tip of the tool, which prevents the lubricant from entering and seriously affect the service life of the tool.

There has been extensive attention to textured surfaces because of their good tribological properties, but the presented state of the art is rather short and incomplete. According to Grutzmacher et al.^2^, textures have the functions of storing debris and lubricants, reducing the actual contact area, and helping to increase the hydrodynamic pressure. Cheng et al.^3^ investigated the friction and wear of different textured surfaces and explored the effects of depth, roughness and load on interfacial friction using a hybrid elastomeric lubrication model. Wei et al.^4^ prepared four types of textured surfaces with different area densities and carried out ball-disc wear tests. The results demonstrated that the textures significantly improved the wear resistance of the substrate material, and the hydrodynamic lubrication effect caused by the texture is the main reason for the friction reduction. Wan et al.^5^ found that surfaces with textures have the smallest COF, and the curve of the COF is more stable than that of the non-texture surface. Costa et al.^6^ analyzed the anti-friction of textured surface in different applications and emphasized the great advantages of laser processing in the preparation of textures. However, Marian et al.^7^ pointed out that textures to control friction and wear in lubricated tribo-contacts is still in the trial-and-error phase.

Further optimization of the lubrication of the textured surfaces can reduce friction and wear, thereby improving energy efficiency and sustainability. Filling the texture with solid lubricants has attracted much attention due to its convenient preparation and self-lubricating properties. Li et al.^8^ deposited soft metal (silver) in the texture, and verified that the surface has no obvious effect on reducing friction at room temperature, while the anti-friction is better at 200 °C, 400 °C and 300 °C. Moreover, the lubricant storage of the texture has a more significant effect on reducing friction at temperatures above 400 °C. Mi et al.^9^ investigated the wear performance of the textured surface filled with WC/Cu solid lubricant. The wear debris formed lubricating film on the surface of Cu phase, and the Cu phase formed self-lubricating film on the hard WC islands. Therefore, the formation of the lubricating film on the wear surface decreased the COF and improved the wear resistance. Huang et al.^10^ used a chemical plating method to deposit Ag/MoS2 solid lubricant into the pits and performed wear tests, the results showed that the COF and wear were greatly reduced because the solid lubricant forms a lubricating film on the surface. Furthermore, Yin et al.^11^ sprayed non-metallic solid lubricant (graphite particles) on a textured surface and concluded that the graphite significantly improved the tribological properties of surface through wear tests. Meng et al.^12^ researched the anti-friction of different types of solid lubricants (CaF_2_, WS_2_ and graphite) in textures. They concluded that the textures filled with either WS_2_ or graphite solid lubricants showed relatively low COF, and the presence of a lubricating film in the friction region protected the specimens from further wear damage. Wang et al.^13^ filled the composite solid lubricant in texture by holding pressure deposition process. The friction tests demonstrated that the texture acts as a lubricant reservoir, gradually releasing the lubricant into the sliding contact region during sliding, and the carbon nanofibers in the composite solid lubricant have the greatest contribution to reducing the friction coefficient. Hua et al.^14^ investigated friction properties of texture filled with polyimide as a solid lubricant in the range of room temperature to 400 °C. The results demonstrated that the COF of the textured surface filled with the flexible lubricant was significantly lower and more stable. Rosenkranz et al.^15^ pointed out in a review that the combination of texture and solid lubricants is a promising approach for achieving surfaces with adjustable friction or wear behavior. However, there are still many opportunities for further improvement and optimization of the technology to maximize the synergies between textures and solid lubricants.

The uniformity of the solid lubricant greatly affects the tribological properties of the textured surface. From the point of view of filling uniformity, an efficient, low-cost and high-uniformity filling method is worthy of interest. The electrostatic flocking technology is considered as a new surface modification technique to change the surface topography of the specimen and increase the surface porosity^16^, thus enhancing the penetration of the lubricant. McCarthy et al.^17^ used Coulombic driving forces to launch short fibers from an electrode plate onto an adhesive-covered substrate, forming a densely array of fibers perpendicular to the substrate. The electrostatic flocking technology is presently used in marine antifouling^18^, solar-driven steam generators^19^, microfluidic chips for self-collecting flow^20^, shock absorbing materials^21^.

In this paper, we propose a anti-friction surface with excellent wetting properties. A mesh texture was first prepared on the surface of high-speed steel, and then nylon fibers were implanted into the grooves of the texture as a solid lubricant by the electrostatic flocking technology. Zeng et al.^22^ reported that the application of vegetable oil in machining has been increasing in recent years due to the emphasis on sustainable manufacturing. Among these, castor oil has excellent lubricating properties due to its higher viscosity. The COF and wear rate of the flocking surfaces were compared under different lubrication conditions by means of a reciprocating wear tester. Then the changes of COF were discussed under different loads. Finally, the wettability and mechanism of high viscosity castor oil on the flocking surface were evaluated. These studies provide a reference for the optimization of wetting and lubrication on tool surface.

## Experimental details

### Materials

HSS is widely used in complex tools because of its excellent thermal hardness and ease of grinding. The preparation of anti-friction surface with HSS substrate is of practical significance to the optimization of tool surface. Therefore, a size of 20 mm × 10 mm × 5 mm is selected as the substrate to prepare the flocking surface. The detailed components (Mass fraction) of HSS are listed in Table 1.

**Table 1:** Main element of HSS.

Element	C	Si	Mn	P	S	Cr	V	W	Mo	Co
Content wt%	1.00 ~ 1.15	≤ 0.65	≤ 0.40	≤ 0.03	≤ 0.03	3.50 ~ 4.50	0.95 ~ 1.35	1.15 ~ 1.85	9.0 ~ 10.0	7.5–8.5

**Figure 1:**
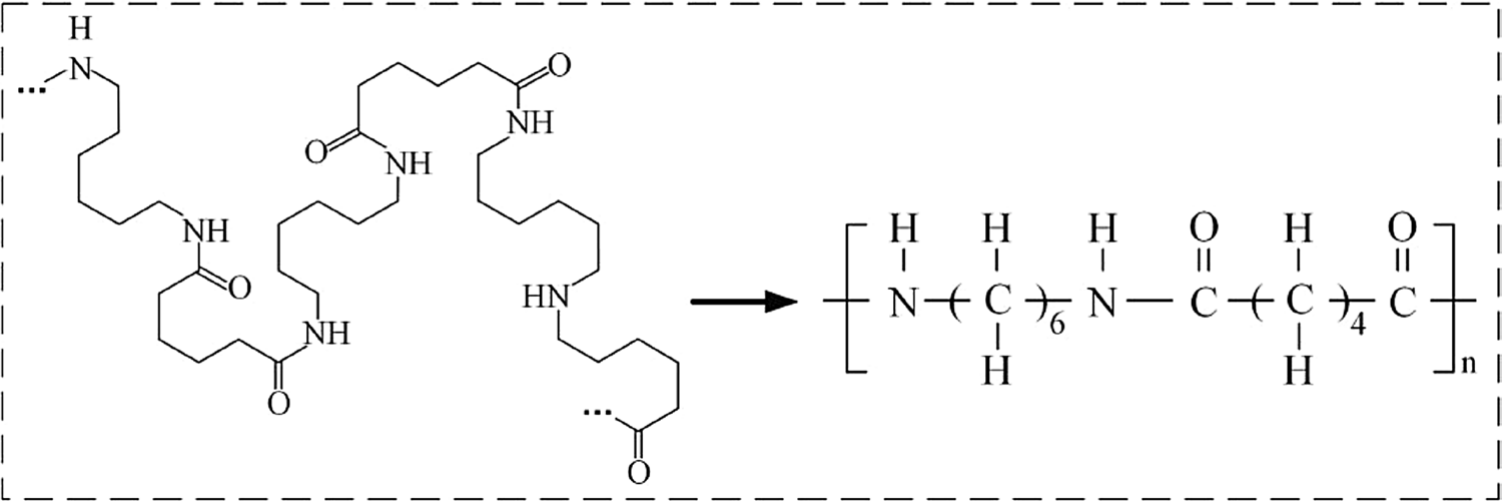
Structural formula of nylon material.

Nylon (Hexamethylene adipamide, PA-66) is selected as a solid lubricant to improve the tribological properties of the surface because of its excellent wear and corrosion resistance, high mechanical strength, and self-lubrication properties. Figure 1 shows the molecular structure formula of nylon material. And nylon fibers (10 μm in diameter and 0.4 mm in length) are supplied by Dongguan Fuhua Co., LTD.

In the cutting process of 304 stainless steel (304 SS), the material tends to bond with the tool surface caused by tool wear due to its poor thermal conductivity. Given the difficult-to-process properties of the material, it is used as the steel ball material for wear tests with HSS, which provides a valuable reference for cutting 304 SS. And Table 2 lists the main elements of the 304 SS.

**Table 2:** Main element of 304 SS.

Element	C	Si	Mn	P	S	Cr	Ni
Content wt%	0.08	1.00	2.00	0.045	0.030	18.0 ~ 20.0	8.0 ~ 11.0

### Preparation of flocking surfaces

**Figure 2:**
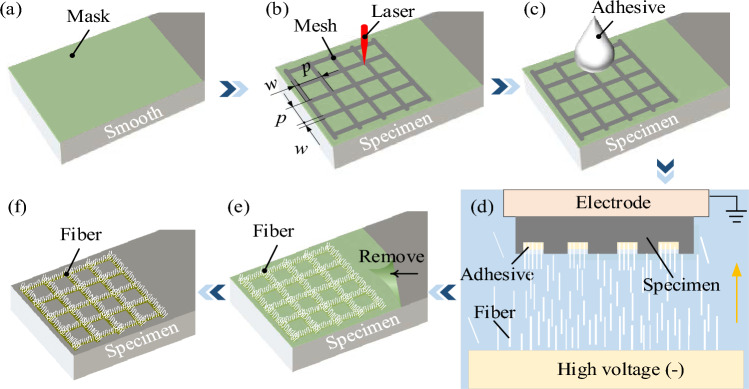
Preparation process of flocking surface: (**a**) paste mask, (**b**) laser processing of texture, (**c**) apply adhesive to the texture, (**d**) electrostatic flocking process, (**e**) mask removal, (**f**) diagram of flocking surface.

Figure 2 shows the preparation process of flocking surface. The flocking surface was pretreated and cleaned with anhydrous ethanol to remove stains on the surface of the specimen. To ensure the quality of the flocking surface, a translucent tape was adhered to the specimen surface to form a mask (Fig. 2a). Then, a H20 laser marking machine (HAN'S LASER, Guangdong, China) was used to prepare the mesh texture. The power of the laser marking machine is 12 W, and its spot is 50 μm. A groove width (*w*) of 0.2 mm (Fig. 2b) had excellent anti-friction characteristics^23^, and Gachot et al.^24^ obtained the best anti-friction effect with the area coverages between 0.3 and 0.5, so setting the pitch (*p*) to 1 mm gave an area coverages of 0.36. The groove depth was set at 0.4 mm after several tests combined with the processing efficiency. The textured surface was then treated with sandpaper to remove the hardened residue that had gathered around the edges of the grooves, and then washed in an ultrasonic cleaner with anhydrous ethanol for 15 min. After drying, 1 μL of flocking adhesive (Anda Huatai New Material Co., LTD, China) was dripped into the grooves along the groove of textured surface using a microinjector (Fig. 2c). And the flocking adhesive is a milky liquid, the main component is polyvinyl acetate, and the viscosity is 10,000–20,000 mPa·s at temperature of 25 °C. Then the specimen was placed on a vibration exciter (SA-JZ020, Wuxi SWO Technology Co., Ltd.) at a frequency of 2 kHz for 1 min to achieve uniform distribution of flocking adhesive. As shown in Fig. 2d, the specimen was fixed in the electrostatic flocking equipment (XT-F06, Jiangsu Xintu Machinery Co., Ltd.), and nylon fibers were polarized and flown vertically to the zero-potential specimen at the top of the flocking box under 120 kV electrostatic voltage. The grooves filled with adhesive will stick nylon fibers that raise to their surface, and this process lasts one minute. The specimen implanted fibers were then dried in a vacuum oven at 80 °C for 4 h to cure the adhesive. Subsequently, the mask on the surface was removed (Fig. 2e), and ultrasonic cleaning was performed with petroleum ether for 15 min to remove the mask residue. Finally, rinse with anhydrous ethanol and deionized water in turn, and dry again before use (Fig. 2f).

### Wear test

Excellent tribological properties can reduce friction and wear when strong sliding friction occurs between two contact surfaces under positive pressure. Therefore, a linear reciprocating wear tester (Rtec 8000) was used to investigate the sliding friction of the specimen surfaces. And the wear test was carried out under the conditions that the environment condition (temperature 25 °C, relative humidity 40%). To provide a reference for the optimization of tool surface, the surface roughness of the specimen was set to be the same as that of the rake face of the tool (broach), that is, Ra = 0.5. And the roughness is measured using a Mitutoyo surface profifiler (type: SJ-210). A 304 SS ball with a diameter of 6.35 mm was used for rubbing against the specimen surface. Considering the test parameters of stainless steel by Yang et al.^25^, and combined with the application background of low-speed cutting, a constant pressure of 80 N was applied to the specimen by the 304 SS ball, reciprocating in the *x*-direction at a frequency of 1 Hz (10 mm/s), the stroke was 5 mm, and the working time was 30 min.

To investigate the tribological properties of the flocking surfaces proposed in this paper, comparative tests are carried out, and the specific arrangements are given in Table 3. The tribological properties of smooth surface, textured surface and flocking surface are compared in dry friction test and castor oil lubrication test, respectively. T1 ~ T3 are dry friction tests, in which the dry friction test of smooth surface is marked as SD, the dry friction test of textured surface is marked as TD, and the dry friction test of flocking surface is marked as FD. Castor oil is selected as the lubricant for the friction test (T4 ~ T6) based on the consideration of environmentally friendly vegetable oil. The smooth surface, textured surface, and flocking surface are marked as SC, TC, and FC, respectively. To meet the sustainable manufacturing requirements, excellent tribological properties under the minimal lubrication deserve to be noticed. Therefore, 3 μL of castor oil is pre-dropped on the friction area, then load and test.

**Table 3:** Experimental arrangement.

Number	Surface	Lubrication	Mark
T-1	Smooth	Dry	SD
T-2	Texture	Dry	TD
T-3	Flocking	Dry	FD
T-4	Smooth	Castor oil	SC
T-5	Texture	Castor oil	TC
T-6	Flocking	Castor oil	FC

To comprehensively evaluate the tribological properties of the surfaces to explain the frictional behavior of the two contact surfaces, the wear rate of the specimens and 304 SS balls are measured. The average value of ten repeatable tests s used as the evaluation data. In addition, the standard deviation of each set of data is used as an error bar to evaluate the repeatability of the test. And in wear measurements, it is most common toreport a wear rate (σ) that follows Archard’s wear equation (Eq. (1)):

1 $$ \sigma = \frac{\nabla V}{{F \cdot S}}, $$

where, ∇*V* is the wear volume, *F* is the applied load, *S* is the sliding distance.

### Wettability test

**Figure 3:**
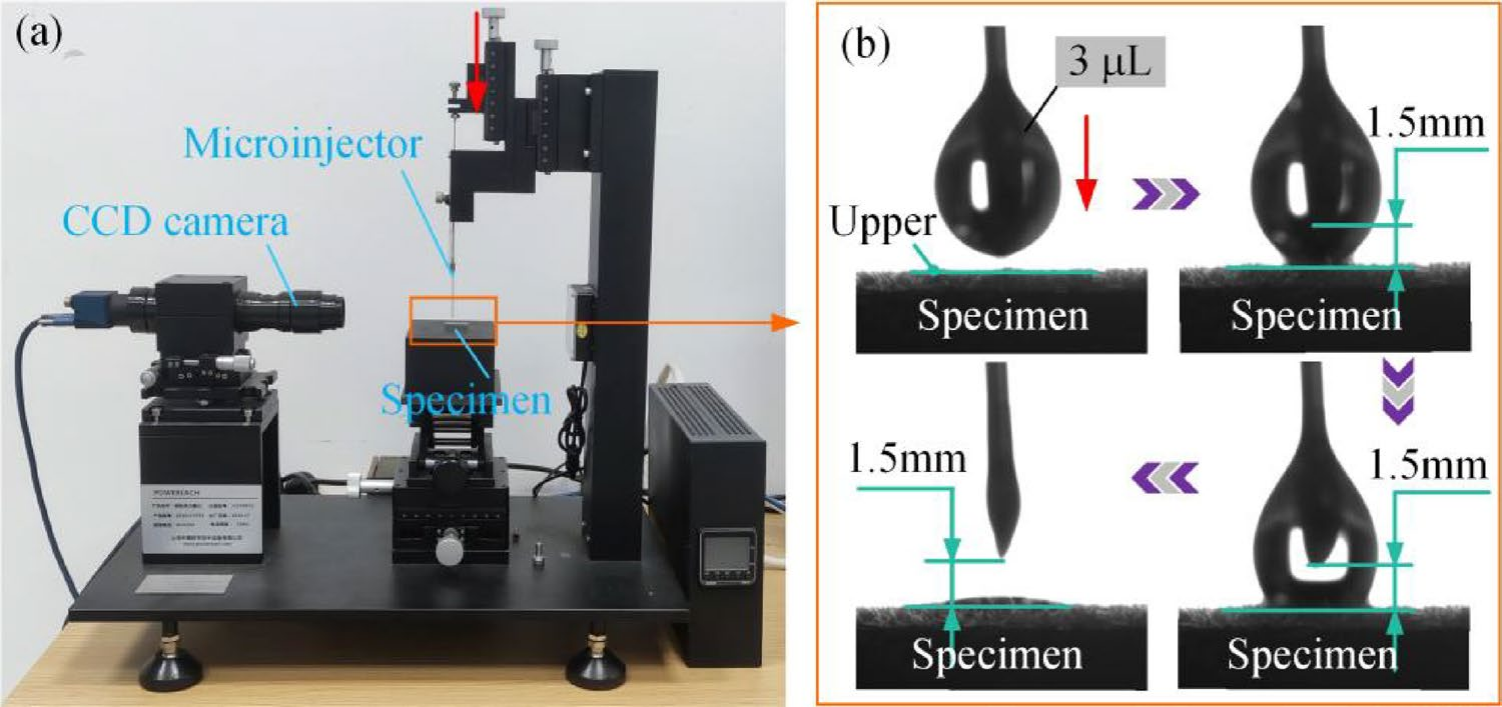
Test system for surface wettability: (**a**) equipment of wettability test, (**b**) test method.

Figure 3 shows the test system and method for researching the wettability of castor oil on different surfaces. As shown in Fig. 3a, a contact angle measuring instrument (JC2000D1, Shanghai Zhongchen Digital Technology Equipment Co., LTD, China) is chosen to photograph the process of oil droplet desorption from the tip of the microinjector to the specimen surface. Before measuring the contact angle, all specimens were washed in an ultrasonic cleaner with ethyl alcohol for 5 min. After rinsed with deionized water, they were dried in a vacuum drying oven (60 °C) for 1 h, and then the contact angle was measured. Figure 3b shows the specific experimental steps. First, a 3 μL droplet was obtained in advance using a microinjector. Then slowly moved the droplet down, and when the needle tip is 1.5 mm away from the specimen surface, the bottom of the droplet just touches the specimen surface. At this point, the morphological changes of the oil droplet completely transferred from the tip of the needle to the specimen surface were recorded at a rate of 10 frames per second.

## Results

### Characterization of flocking surface

**Figure 4:**
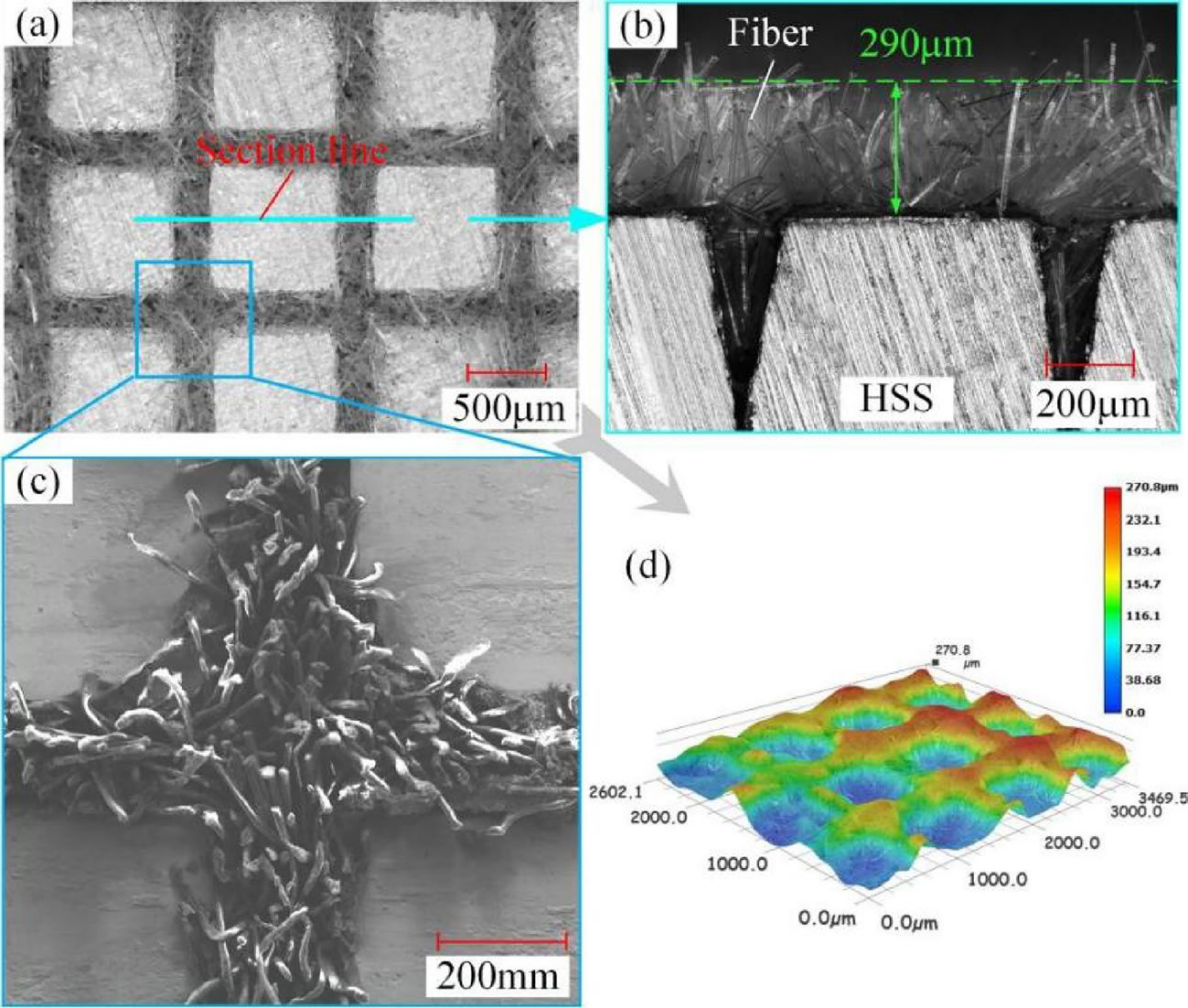
Characterization of flocking surface: (**a**) flocking surface at 100 ×, (**b**) cross section at 200 ×, (**c**) partial enlarged view at 300 ×, (**d**) 3D morphology of flocking surface at 100 ×.

The morphology of the flocking surface is characterized in Fig. 4, and Fig. 4a shows the flocking surface photographed with a high-speed digital camera (Type: KEYENCE VW-9000) at a magnification of 100 ×. To reflect the state of the nylon fiber implanted in the groove in detail, the cross section of the specimen was taken at 200 × according to the direction of tangent line in Fig. 4. As given in Fig. 4b, the size of fibers protruding from the surface is about 290 μm. The length of the nylon fibers selected was 400 μm, which means that the implantation length in the groove is about 110 μm. Figure 4c shows a partial enlarged view of the meshed intersection at a magnification of 300 ×. In addition, 3D morphological characterization of the flocking surface (Fig. 4d) shows that the nylon fibers are distributed on the HSS surface as a whole, forming a connected and bulged mesh.

Table 4 lists the actual size of the texture and the thickness of the nylon coating after drying. The actual value is obtained by a high-speed digital camera to measure the dimensions of five different positions on the same specimen, and then measuring the three specimens separately.

**Table 4:** Design and actual dimensions.

Type	Item	Design (mm)	Actual (mm)	Average (mm)
Texture	Width (*w*)	0.2	0.188–0.221	0.212
	Pitch (*p*)	1	0.991–1.011	1.003
	Depth	0.4	0.373–0.410	0.395
Coating	Thickness	0.3	0.265–0.327	0.290

### Coefficient of friction

**Figure 5:**
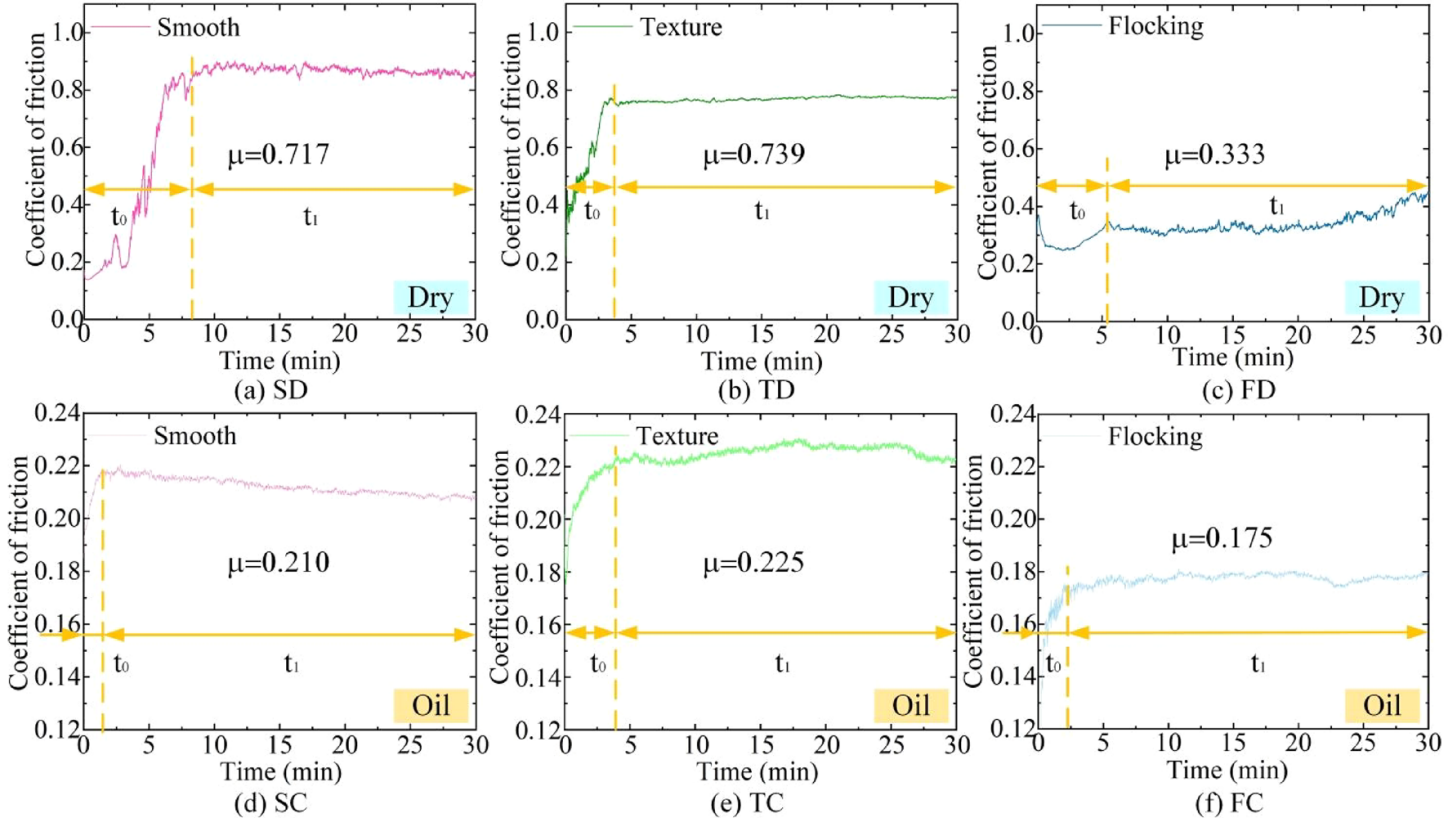
COF of three surfaces under different lubrication conditions.

Figure 5 shows the curves of COF for three surfaces under different lubrication conditions. The wear test is carried out ten times under each condition to ensure the repeatability and reliability of the data, and one of the ten groups of tests is randomly selected for characterization. The values of COF marked in the figures are the average COF of the ten groups of tests. In addition, the curves of COF is divided into two stages with yellow dashed lines, namely, the initial stage t_0_ and the stable stage t_1_. It is used to discuss the tribological properties of each surface at different stages. The datasets used and/or analysed during the current study available from the corresponding author on reasonable request.

In the dry friction condition (Fig. 5a–c), TD has the largest average COF of 0.739, which is 3.1% higher than that of SD. However, the COF of SD is significantly smaller than that of TD during the initial stage (t_o_) and higher than that of TD during the stable stage (t_1_). The COF of FD reduces to 0.333 by implanting nylon fibers into the groove, which was 54.9% lower than TD and 53.6% lower than SD. Moreover, FD decreases first and then increases slowly in the t_0_ stage, and the variation range is small. This indicates that the addition of nylon fibers reduces the overall COF, and also alleviates the large frictional force caused by the shearing of the two friction surfaces during the initial stage of the material.

Under the lubrication conditions of castor oil (Fig. 5d–f), the overall trend of COF is the same as that of dry friction, with the largest TC (0.225), followed by SC (0.210). FC (0.175) is the smallest, 22.2% lower than TC and 16.7% lower than SC. The difference is that the curve of COF of the textured surface at t_1_ is higher than that of the smooth surface. Because the addition of castor oil suspends some of the debris generated by friction in the frictional region, weakening the ability of the texture to store debris. Combined with the cutting phenomenon of the material by the sharp edges of the texture, the COF of textured surface is higher than that of the smooth surface. In addition, the presence of castor oil caused friction to move more rapidly from t_o_ into t_1_, this phenomenon is more obvious on the flocking surface. The synergistic effect of nylon fiber and castor oil improve the lubrication, which enhances the tribological properties of the surface.

### Wear

**Figure 6:**
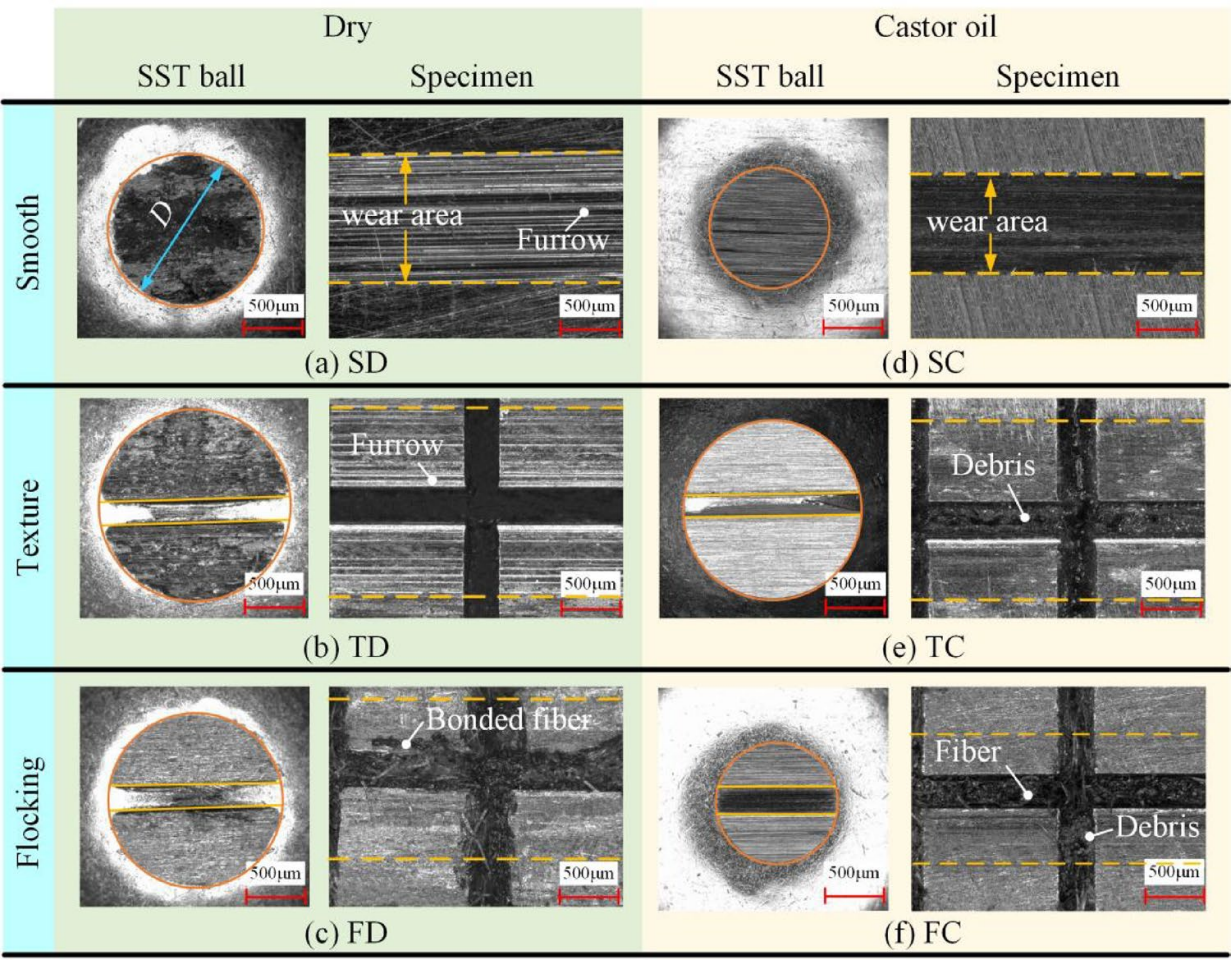
Wear scar of specimen and SST ball.

Figure 6 displays the micrographs of the wear scar on the specimen and the 304 SS after the test. The 304 SS balls and specimen were washed with deionized water, and then washed in ultrasonic cleaning machine containing anhydrous ethanol for 15 min to remove debris and oil on the surface. Then the wear scars of the steel balls are observed using a high-speed digital camera at a magnification of 150 ×, while the wear scars on the specimen surface are observed at 200 ×. Under dry friction condition, the surfaces of SS ball are rough and have many defects, among which the surface quality of FD is the best and the wear-scar lines are clear. TD is second, but the wear-scar lines is unrecognizable. SD has the worst worn surface and has the most serious defects.

The wear scars on the specimen surfaces show that the SD has a large number of furrows. These furrows are mainly caused by abrasive wear, which comes from debris generated in the process of friction between the two contact surfaces. TD also has furrows, but to a lesser extent, where the presence of texture stores debris and reduces surface damage. FD has no obvious furrows appear, but there are nylon fibers at the edge of the grooves that cannot be remove by ultrasonic cleaning. It indicates that the nylon fibers were squeezed as well as bonded to the specimen surface during repeated friction.

The surface quality of the SS ball improves obviously under castor oil lubrication conditions. There are a few scratches on the surface of the SC, but no significant large-area damage compared with the dry friction. The surface of TC is clean, with no scratches. FC has a small number of scratches, which indicates that FC is slightly less capable of storing debris than TC. By observing the surface of the specimen, the wear scars on the specimen surface are significantly improved by the lubrication of castor oil. On the surface of TC and FC, there is no obvious presence of scratches and wear scars, but a large number of debris exists in the grooves of the texture.

**Figure 7:**
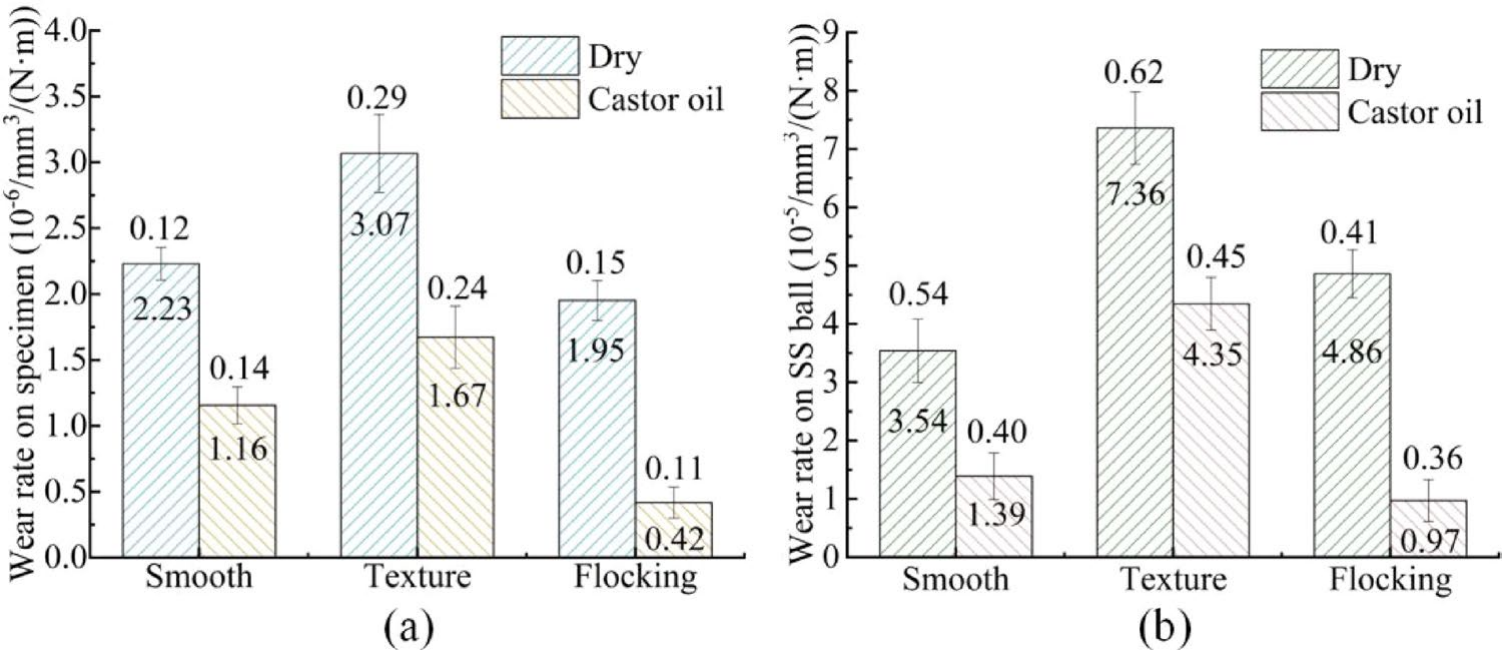
Wear rate: (**a**) wear rate on specimen, (**b**) wear rate on 304 SS.

Figure 7 respectively shows the wear rate of the specimen and the 304 SS ball after the test, which is calculated according to Eq. (1). The wear volume of the specimen is calculated using the mass loss and the density of the material. The mass loss is obtained by the electronic balance (ZG-TP203) with an accuracy of 1 mg. The wear volume of the 304 SS is calculated by the diameter of the wear scar. The datasets used and/or analysed during the current study available from the corresponding author on reasonable request.

It can be seen from Fig. 7a,b that the wear rate on the surface of the specimen and 304 SS ball changes in the same trend. The wear rate of the two contact surfaces is the highest with the texture, but the wear rate of both of them decreases after the nylon fiber implantation. And the wear rate of flocking surface is greatly reduced in the application of castor oil. Compared with smooth surfaces, flocking surfaces reduce wear rate by 12.6% under dry friction and 63.8% under castor oil. The above results demonstrate that nylon fiber enhances the wear resistance of the contact surface under castor oil lubrication. Furthermore, the low wear rate of HSS surface is of great significance to the tool life.

### COF under different loads

**Figure 8:**
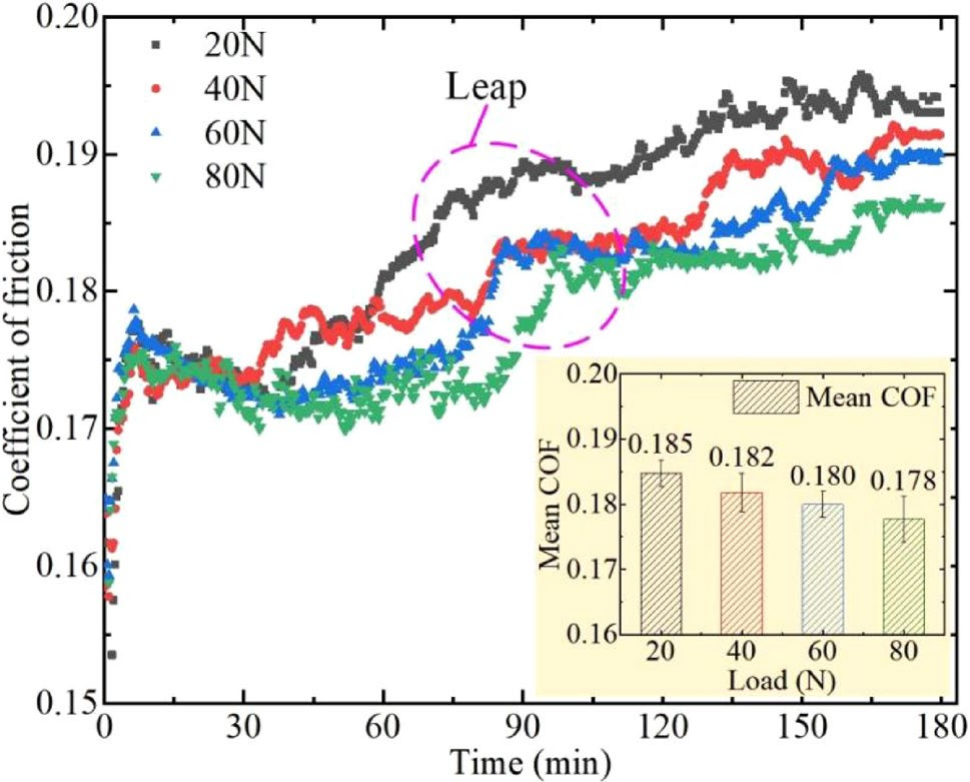
COF of flocking surface under different loads.

Figure 8 shows the COF and average COF of the flocking surface under different loads. Previous experiments have proved that the flocking surface has excellent anti-friction properties. To further investigate the performance of the flocking surface under different loads, a 3-h wear test is performed lubricated by castor oil. The datasets used and/or analysed during the current study available from the corresponding author on reasonable request.

In the first 30 min, the COFs under various loads are approximate. After 30 min, the COF of 20 N and 40 N increases rapidly, while the COF of 60 N and 80 N did not increase until 45 min later. At all loads,the higher the load applies, the smaller the COF is. At about 90 min, the COF will show a jumping growth, with the increase of load, the time of jumping growth will be delayed correspondingly, that is, the longer the time to maintain a small COF. Combined with the average COF given in the bar chart, the maximum COF is 0.185 at 20 N, and the minimum COF is 0.176 at 80 N. The minimum COF is reduced by 3.8% compared with the maximum COF. It is concluded that the flocking surface has the best anti-friction property when the load is applied to 80 N, but the anti-friction property is not obvious with the small reduction of the load.

## Discussion

### Influence on tribological properties

**Figure 9:**
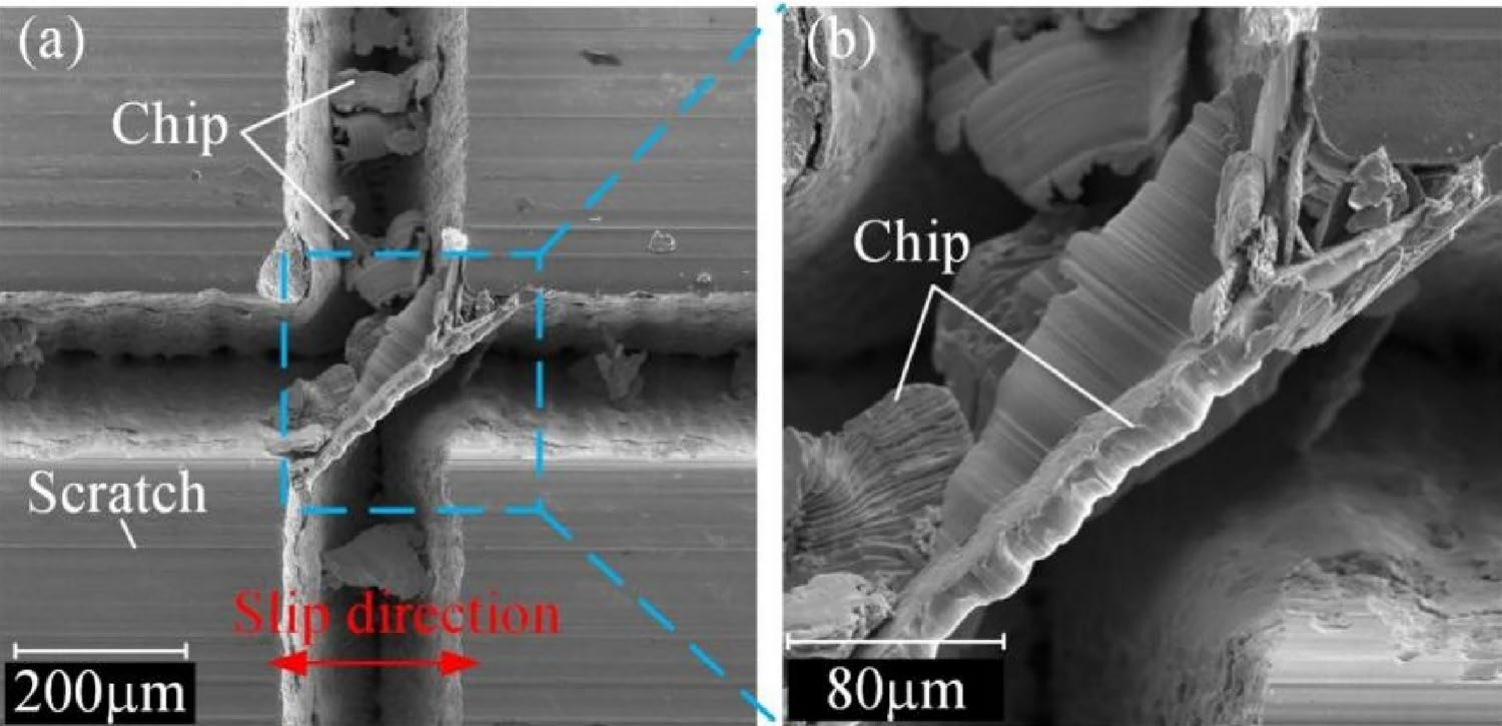
Cutting phenomenon on textured surface, (a) SEM at 300 ×, (b) SEM at 1000 × .

It is found that the texture prepared in this experiment increased the COF and wear on the surface by comparison tests with the smooth surface and the textured surface. Figure 9 exhibits the micrographs of the textured surface after the wear tests, which is taken by a scanning electron microscope (SEM). At a magnification of 300 ×, a large number of chips appear in the grooves, and mainly distribute in the longitudinal grooves perpendicular to the sliding direction (Fig. 9a). These chips are caused by the 304 SS ball being cut by the longitudinal edges of the texture. Figure 9b shows the chips generated by the severe cutting phenomena at the edge of texture (1000 ×). These large chips were accompanied by the removal of excess materials, which increased the friction force and eventually the COF.

**Figure 10:**
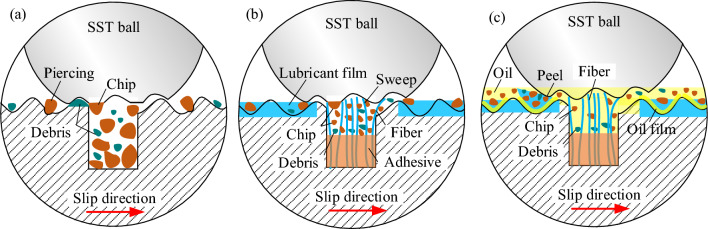
Mechanism of anti-friction on surfaces: (**a**) textured surface; (**b**) flocking surface; (**c**) flocking surface under the lubrication of castor oil.

As shown in Fig. 10a, a large area of chips will pierce the specimen surface with the relative extrusion of the two contact surfaces or cause surface damage with the peeling of chips. At the same time, serious cutting phenomenon causes excessive removal of material on the steel ball and produces a large diameter of wear scar. However, the existence of the texture stores the chips from the 304 SS ball and the debris from the specimen material during the friction process, preventing them from damaging the two contact surfaces and reducing scratches, improving the quality of the two friction surfaces.

As displayed in Fig. 10b, when the nylon fibers are implanted into the texture, the nylon fibers located near the groove edge will play a cushioning role in the material shear caused by the relative friction between the two surfaces, thus slowing down the cutting phenomenon at the textured edge. The shear strength of nylon material (25.2–27.8 MPa) is far less than 304 SS (187.2–249.6 MPa), so the nylon fiber in the fracture along the sliding direction to overcome the force is far less than 304 SS, thus reducing the friction force. In addition, the broken fibers are dragged by the contact surfaces and squeezed on the microscopic uneven surface of the specimen to form a solid lubrication film^26^, which plays a role in repairing the surface and making the surface flat and smooth. The flat surface reduces the shear between the bulges in the contact area with the 304 SS ball and reduces the size of chips and debris, thus reducing friction. These tiny fragments are squeezed into a lubricating film made of nylon fibers, preventing surface damage caused by direct interaction with the metal surface. And the nylon fiber has high elastic recovery ability^27^, making it act as a brush in the friction process, timely remove the adsorption on the ball surface of the tiny debris and avoid its entry into the friction area caused by abrasive wear. In conclusion, the nylon fibers implanted in the groove can significantly improve the tribological properties of the surface, not only reducing the friction coefficient, but also effectively improving the surface damage of the specimen, and decreasing the diameter of the wear scar on the 304 SS ball.

Under the lubrication of castor oil (Fig. 10c), the oil film on the surface improves the friction state, and the relative sliding of the two contact surfaces removes worn debris and improves the surface quality. During the wear process of flocking surface, the gap between the two surfaces is increased due to the existence of solid lubrication film formed by the extrusion of nylon fiber and liquid oil film formed by castor oil, so that the contact volume of microscopic convex peaks on the two surfaces is reduced, and the volume of material shear at the convex peaks is also reduced, which ultimately leads to the reduction of COF. In addition, before the friction begins, the castor oil will first saturate the surface and form an oil film. When the nylon fibers broken in the frictional process are squeezed on the surface to form a solid lubrication film, there will be a layer of oil film between the solid lubrication film and the metal substrate, which reduces the adhesion strength of the nylon material and the substrate. As the numerous debris are extruded into the solid lubrication film, the accumulation of these debris increases the resistance between the ball material and the specimen surface. With resistance increases, the surface of the 304 SS ball pulls the nylon material away from the substrate. A new solid lubricant film is then re-formed during friction, and this repeated formation and peeling of the lubricant film removes large amounts of debris, further reducing surface wear. This is also the reason why nylon fibers adhere to the surface after dry friction in Fig. 6, while no nylon fibers adhere to the surface under oil lubrication. Kalin et al.^28^ researched the tribological behavior of solid lubricant and lubricating oils. It was confirmed that the solid lubricant compacted and deformed in the friction region to form a wear-resistant and low-shear film, which also caused the boundary film to thicken. In a word, the existence of both the solid lubricating film and the oil film at the friction region increases the gap between the two contact surfaces, which makes the lubricating film thicker and more fully lubricated. Also, they can peel off the solid lubricating film carrying a large amount of debris under the action of small shear force, thus reducing the occurrence of abrasive wear, so that the COF is reduced and the wear-scar diameter of the 304 SS ball is decrease.

According to the binomial law of friction^29^, sliding friction is a process to overcome the adhesion and molecular attraction of convex peaks on the surface, and friction is the sum of mechanical action and molecular action resistance, and the comprehensive COF formula is given in formula (2).

2 $$ \mu = \frac{\alpha A}{F} + \beta , $$

where, α and β are the coefficients determined by the physical and mechanical properties of the surface material respectively, A is the actual contact area, and *F* is the applied load. For further discussion, the formula of the applied load *F* is introduced:

3 $$ F = AP, $$

where, *P* is the normal phase load per unit area. Combined with formula (2) and (3), the expression of the comprehensive COF is obtained:

4 $$ \mu = \frac{\alpha }{P} + \beta . $$

According to formula (4), when the *P* increases, the COF decreases. This is consistent with the experimental results in this paper, that is, the COF decreases with increasing pressure under different applied loads. Furthermore, nylon fibers under small load, the degree of plastic deformation are small, nylon fibers are dragged along the friction region by two contact surfaces. Srinath et al.^30^ verified that when nylon slides on the metal surface, it will form a lubricating film and play a role in shielding the hard metal protrusion in the friction region. With the continuous increase of load, the plastic deformation of nylon fiber increases until it is squeezed into the substrate to form a lubricating film, which makes the specimen surface more smooth and flat, and the flat surface reduces the COF.

### Influence on wettability

**Figure 11:**
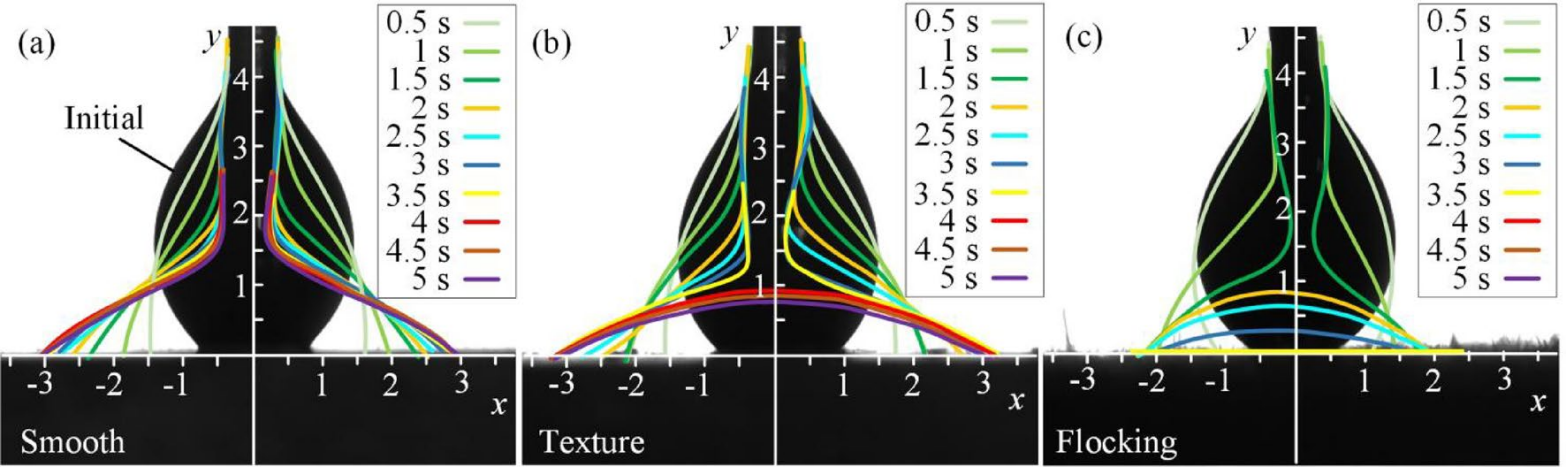
Wettability of droplet on different surfaces: (**a**) smooth surface; (**b**) textured surface; (**c**) flocking surface.

Figure 11 shows wettability of oil droplet on three surfaces, using 3 μL of castor oil drops on the specimen to observe the contour change within 5 s. The contours of droplets at different times are drawn by curve fitting with different colors. The way that lubricating oil exists in the friction pair as an important means of friction reduction deserves attention. Therefore, the wettability of green vegetable oil (castor oil) with high viscosity on the flocking surface is studied, and the influence of different surfaces on wettability is characterized by observing the infiltration of oil drops on the surface. Each group of tests is carried out 5 times, and a group of tests is randomly selected for characterization.

When the oil droplets were in contact with a smooth surface (Fig. 11a), the edge of the bottom spread rapidly along the *x*-axis. At 0.5 s, the edge of the bottom spread to about 1.5 mm, and after 1.5 s, the spread rate slowed down. By the 5th s, the edge of the bottom of the oil drop has spread to a position of 3 mm, while the top drops to a position of 1.5 mm and remain attached to the tip of the microinjector. The spread rate of oil droplets on the textured surface (Fig. 11b) for the first 0.5 s was similar to that on the smooth surface, and the edge of the bottom spreading to 1.5 mm. During the period of 1 s–1.5 s, the spreading of oil droplets in the *x*-axis direction stops briefly, while the *y*-axis direction is still falling, because the oil droplets enter the groove and flow in the groove. After 1.5 s, the edge of the oil drop begins to move along the x-axis again, but at a slower rate. Until the 4th s, the oil drop completely separated from the tip of the microinjector, but the contour changed little after the oil drop separated. According to the research of Wang et al.^31^, without considering the influence of temperature, liquid flow in the groove is mainly affected by capillary force *F*_γ_, liquid viscous resistance *F*_η_ and additional stress *F*_s_ on droplet surface, as shown in formula (5).

5 $$ m\frac{{\partial^{2} x}}{{\partial t^{2} }} = F_{\gamma } + F_{s} - F_{\eta } . $$

As the droplet height decreases, the additional stress *F*_*s*_ on the surface decreases, and formula (6) is satisfied, the liquid stops flowing on the surface.

6 $$ F_{\eta } = F_{\gamma } + F_{s} . $$

When the oil drops on the flocking surface for 0.5 s, the bottom edge spreads slowly to 1 mm (Fig. 11c). This is because when the droplet is just in contact with the surface, the contact line is pinned on fibers, which hinders the spreading of the droplet on the surface. This conclusion is in line with the investigation of Matthew et al.^32^ that contact angle hysteresis is due to contact lines pinning on microscopic irregularities on the surface. Then the contour of the droplet decreases rapidly in the *y*-axis direction, but it no longer extends outward when it spread 2 mm along the *x*-axis direction. At 2 s, the droplet completely separated from the tip of the microinjector, and at 3.5 s completely infiltrated the flocking surface. Based on previous research^33^, this is attributed to the fact that the gap between the fibers in the groove provides extra capillary force *F*_γ_, which enables the liquid to infiltrate rapidly into the gap between the fibers in the groove. This facilitates the prelubrication and storage of the lubricant on the surface and continuously provides sufficient lubricant for the contact area during the friction process to improve the tribological properties of the surface.

## Conclusion

This paper used electrostatic flocking technology to implant nylon fibers into grooves on the texture surface to solve the problem of insufficient lubrication at friction region. The tribological properties of smooth surface, textured surface, and flocking surface were studied under dry friction and oil-lubricated condition. In addition, the wettability of the three surfaces was also studied. The details are as follows:

1. The texture increases the COF and wear due to the cutting phenomenon of its edges, while the implantation of nylon fibers in the grooves provides effective cushioning and decreases the cutting of the material. This reduces the COF by 53.6% in dry friction conditions and 16.7% in oil-lubricated conditions.
2. The broken fibers are extruded in the contact surface to form a solid lubricating films. The existence of solid lubricating films fill the microscopic uneven area of the surface so that the shear between the convex peaks becomes less, so as to reduce friction, and avoid the damage to the surface by large debris. The flocking surfaces reduce wear rate by 12.6% under dry friction and 63.8% under castor oil compared with smooth surfaces.
3. It is the combination of the solid lubricating film formed by the nylon fiber and the castor oil that increases the gap between the contact surfaces and makes the lubrication more adequate. In addition, due to the existence of a layer of oil film between the solid lubricating film and the substrate, the solid lubricating film that stores a lot of debris is easier to peel from the surface and take away debris.
4. The flocking surface has a strong adsorption capacity for high-viscosity castor oil. Owing to a large number of pores between the fibers, the capillary forces in the grooves are increased, so that the liquid will quickly penetrate and be stored in the groove, providing a continuous lubricant for the friction region during the friction process.

## Data Availability

The datasets used and/or analysed during the current study available from the corresponding author on reasonable request.
